# Magnetic resonance imaging findings of pulmonary sclerosing pneumocytoma: a case report and literature review

**DOI:** 10.3389/fonc.2023.1158328

**Published:** 2023-09-01

**Authors:** Yang Li, Li Yang, Xiaolong Gu, Yaning Wang, Huiyan Deng, Hui Feng, Ning Zhang, Mingbo Wang, Qi Wang, Gaofeng Shi

**Affiliations:** ^1^ Department of Computed Tomography and Magnetic Resonance Imaging, The Fourth Hospital of Hebei Medical University, Shijiazhuang, Hebei, China; ^2^ Department of Pathology, Hebei Medical University Fourth Affiliated Hospital and Hebei Provincial Tumor Hospital , Shijiazhuang, Hebei, China; ^3^ Department of Thoracic Surgery, Hebei Medical University Fourth Affiliated Hospital and Hebei Provincial Tumor Hospital, Shijiazhuang, Hebei, China

**Keywords:** pulmonary sclerosing pneumocytoma, pulmonary, magnetic resonance imaging, computed tomography, case report

## Abstract

**Background:**

Pulmonary sclerosing pneumocytoma (PSP) is a rare lung tumor that is mostly isolated and commonly reported among middle-aged East Asian women. Recently, Immunohistochemistry (IHC) analysis has suggested that PSP is of primitive epithelial origin, most likely derived from type II alveolar air cells. Patients with PSP are generally asymptomatic and usually detected for other unrelated reasons during routine imaging. Several studies have already investigated the computed tomography (CT) features of PSP and their correlation with pathology. Magnetic resonance imaging (MRI) is a radiation-free imaging technique with important diagnostic value for specific pulmonary nodules. However, very few case reports or studies focus on the MRI findings of PSP.

**Case report:**

We reported a case of an asymptomatic 56-year-old female with a solitary, well-defined soft-tissue mass in the lower lobe of the left lung. The mass showed iso-to-high signal intensity (SI) than muscle on T1-weighted image (T1WI) and T2-weighted image (T2WI) and a much higher SI on fat-suppressed T2WI, diffusion-weighted image, and apparent diffusion coefficient image. Contrast-enhanced fat-suppressed T1WI revealed noticeable inhomogeneous progressive enhancement throughout the mass. The mass revealed early enhancement without a significant peak, followed by a plateau pattern on dynamic contrast-enhanced MRI images. The patient underwent left basal segmentectomy *via* thoracoscopic surgery. Histopathology and IHC results of the surgical specimen confirmed that it was a PSP. We concluded that the MRI findings of PSP might adequately reflect the different components within the tumor and aid clinicians in preoperative diagnosis and assessment. To the best of our knowledge, this is the most comprehensive case report on the MRI findings of PSP.

**Conclusion:**

The MRI findings of PSP correspond to its histopathological features. Here, we present a case of PSP with the most comprehensive MRI findings, emphasizing the importance of multiple-sequence MRI in diagnosing PSP.

## Background

Pulmonary sclerosing pneumocytoma (PSP), formerly known as pulmonary sclerosing hemangioma, is a rare benign or low-grade malignant lung tumor that commonly occurs in middle-aged Asian women. It was first reported by Hubbell and Liebow (1) in 1955. Initially, it was thought to originate from pulmonary blood vessels, so it was named “sclerosing hemangioma.” With advancements in pathology and immunohistochemistry (IHC), PSP has been shown to be of primitive epithelial origin, most likely from type II alveolar pneumocytes (2). Pathologically, PSP usually has four main histological patterns, namely solid, papillary, sclerotic, and angiomatous areas. Computed tomography (CT) is the primary imaging method for detecting and diagnosing PSP, but it is challenging to diagnose and differentiate PSP preoperatively (3). PSP usually presents as a well-defined, round soft-tissue mass with obvious and heterogeneous enhancement (4). However, few studies have described the magnetic resonance imaging (MRI) findings of PSP, and few cases have been reported. In this report, we presented a surgically confirmed PSP and analyzed its CT and MRI findings in detail.

## Case presentation

A 56-year-old asymptomatic female was referred to The Fourth Hospital of Hebei Medical University (Hebei, Shijiazhuang, China) after a routine physical chest X-ray screening revealed a nodule in the left lower lung field. Physical examination showed no obvious positive signs. The patient had no history of surgery or trauma. There was no family history of hereditary diseases. The patient was presented with no respiratory symptoms and had no history of smoking or alcohol consumption. The patient was diagnosed with hypertension five years ago and has been maintaining normal blood pressure by taking medicine. There was no record of any past surgical procedures or traumatic incidents, and the patient had no family history of hereditary diseases. The results of blood tests and tumor marker analysis were within the normal range.

**Figure 1:**
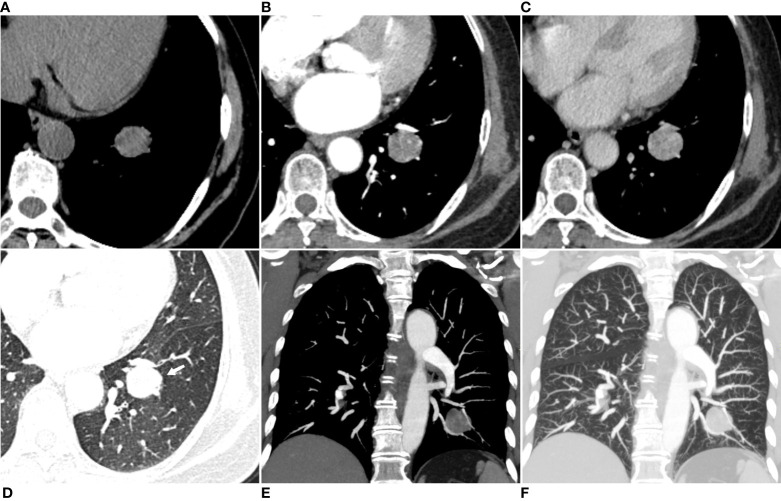
Axial pre-contrast CT image (mediastinal window setting) **(A)** revealed a solitary well-defined soft-tissue mass in the size of 3cm×3 cm×3 cm, with some low-attenuation areas in the lower lobe of the left lung. Axial arterial phase CECT image (mediastinal window setting) showed a heterogeneous and obvious enhancement of the mass, with multiple focal areas of marked enhancement visible within the tumor **(B)**. Axial venous phase CECT image (mediastinal window setting) showed further enhancement of the mass with irregular multiple focal low-attenuation areas internally **(C)**. Axial lung window setting image showed a clear interface between the tumor and the lung, with unremarkable low-attenuation visible in the distal lung tissue (arrow) **(D)**. The coronal CECT images in the mediastinal window setting **(E)** and lung window setting **(F)** showed that the tumor margin closely compressed the adjacent pulmonary artery and vein branch vessels.

The patient underwent pre-contrast and dual-phase (35 seconds in the arterial phase and 70 seconds in the venous phase) contrast-enhanced CT (CECT) scans of the chest five days before the operation. Pre-contrast CT image revealed a well-defined round soft-tissue mass in the lower lobe of the left lung, with a size of about 3 cm × 3 cm × 3 cm (**Figure 1A**). No evidence of cavitation, fat, or calcification was found. The mass showed significant heterogeneous enhancement with an “adherent vessel sign” on the proximal side of the hilum in the arterial phase. Multiple areas of marked enhancement of the lesion were observed within the tumor (**Figure 1B**). In the venous phase, the enhancement further increased with multiple foci of hyperenhancement (**Figure 1C**). The CT value of the mass on the pre-contrast CT image was 26.52 ± 8.24 Hounsfield units (HU). The mass was markedly and heterogeneously enhanced, and its CT number increased to 79.12 ± 28.12 HU in the arterial phase and 96.87 ± 19.23 HU in the venous phase. Axial lung window setting image showed a clear interface between the tumor and the lung (**Figure 1D**). Coronal CECT images clearly depicted the relationship between the mass in the lung tissue and bronchovascular bundles (**Figures 1E, F**). No pleural effusion or enlarged mediastinal lymph nodes were found.

The MRI scan was performed with a 3.0 T MRI scanner (MAGNETOM Skyra, Siemens Healthcare, Erlangen, Germany) using a body phased-array coil (18 elements). The patient was scanned in the supine position throughout the examination. Pre-contrast MRI sequences included in-phase T1-weighted image (T1WI), out-phase T1WI, T2-weighted image (T2WI), fat-suppressed T2WI, diffusion-weighted imaging (DWI; b value =0 s/mm^2^ and 800 s/mm^2^). Then, the patients underwent a pre-contrast volume interpolated breath-hold gradient-echo examination (VIBE), followed by the free-breathing dynamic contrast-enhanced (DCE)-VIBE sequence, started 20 seconds after a bolus injection of gadopentetate dimeglumine (Magnevist, Bayer HealthCare) at a dosage of 0.1 mmol/kg through a cubital vein at a rate of 3 mL/s by an MRI-compatible automated injector pump, followed by a saline chase of 20 ml at the same injection rate. The free-breathing DCE-VIBE sequence included 37 phases, with a total imaging time of 250 seconds.

**Figure 2:**
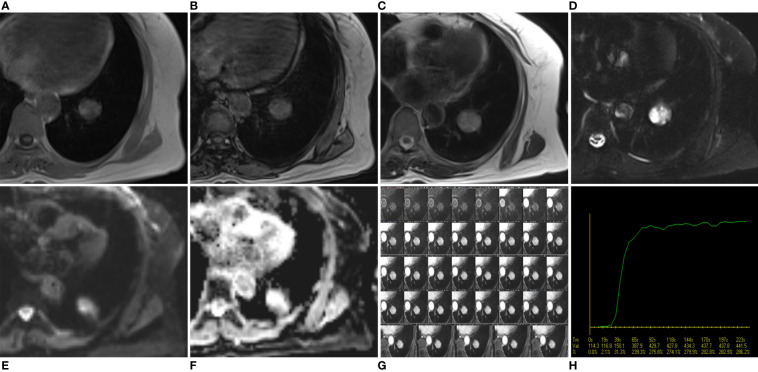
Axial in-phase T1WI image **(A)** and out-phase T1WI image **(B)** showed a round mass with iso-to-high SI compared with the thoracic muscle and mixed with some low SI foci within the mass. On the out-phase T1WI image, no areas of obvious signal reduction were found within the tumor compared to the in-phase T1-weighted image. On axial T2WI image **(C)**, the tumor showed iso-to high SI with some small low SI foci, while on axial fat-suppressed T2WI image **(D)**, the tumor showed a much higher SI. On axial DWI image **(E)** and ADC image **(F)**, the mass showed a much higher SI, and the ADC value was (2.36 ± 0.22)×10^-3^mm^2^/s, suggesting that the mass was a benign tumor. DCE-MRI images **(G)** and corresponding time intensity curve of the SI **(H)** after intravenous injection of Gd-DTPA show early enhancement without obvious peak point, and subsequent plateau pattern.

On the in-phase T1WI image, the mass presented iso-to-high signal intensity (SI) than that of muscle and mixed with some low-SI foci within the mass (**Figure 2A**). On the out-phase T1WI image, the mass still showed iso-to-higher SI with no areas of SI reduction within the tumor compared to the in-phase T1WI image (**Figure 2B**). On the T2WI image, the mass also presented iso-to-higher SI than that of muscle and with some small low SI foci (**Figure 2C**). On the fat-suppressed T2WI image, the mass exhibited heterogeneous signal intensity (SI), characterized by predominantly high SI with scattered regions of low SI within the tumor (**Figure 2D**). On the fat-suppressed T2WI image, the SI was not reduced and became relatively higher (**Figure 2D**). On the DWI image (**Figure 2E**) and the apparent diffusion coefficient (ADC) image (**Figure 2F**), the mass exhibited significant high SI and a measured ADC value of (2.36 ± 0.22) × 10^−3^ mm^2^/s. These findings suggested that the mass was more likely to be a benign tumor rather than a malignant one. Contrast-enhanced fat-suppressed T1WI revealed noticeable marked heterogeneous progressive enhancement throughout the mass (**Figure 2G**). The mass showed early enhancement on DCE-MRI images without a significant peak, followed by a plateau pattern (**Figures 2G, H**).

**Figure 3:**
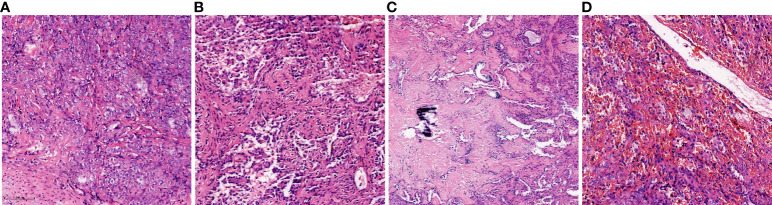
Histopathology of the present case of PSP. It was composed of four major histologic patterns: **(A)** solid pattern (20%, Hematoxylin and eosin, ×20), **(B)** papillary pattern (10%, Hematoxylin and eosin, ×20), **(C)** sclerotic pattern (40%, Hematoxylin and eosin, ×20), and **(D)** hemangiomatous pattern (30%, Hematoxylin and eosin, ×20).

**Figure 4:**
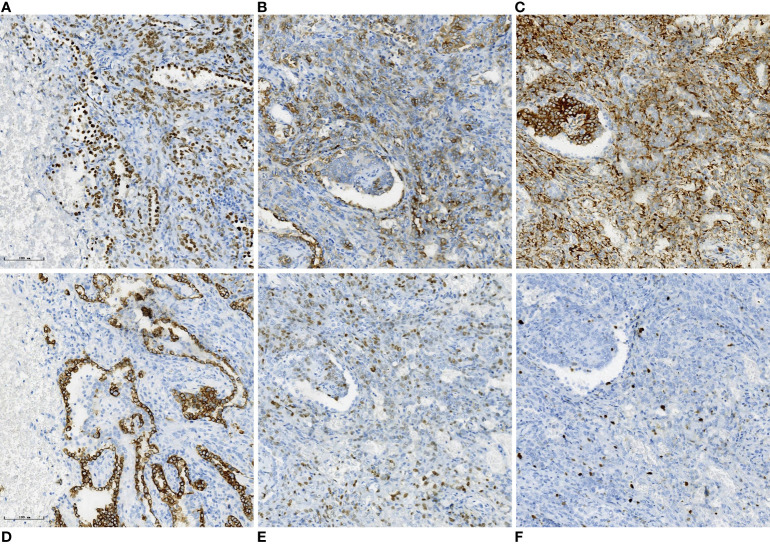
Immunohistochemical results. Immunohistochemical staining (×20) showed that the cuboidal surface cells and round stromal cells the of the tissue are positive for thyroid transcription factor-1 (TTF-1) **(A)**, epithelial membrane antigen (EMA) **(B)** and Vimentin **(C)**. AE1/AE3 was positive in cuboidal surface cells but negative in round stromal cells **(D)**, while progesterone-receptor (PR) exhibited a negative result in cuboidal surface cells but a positive result in round stromal cells **(E)**. The Ki-67 index was below 2% **(F)**.

The mass was surgically resected two days after the MRI examination. Local resection was infeasible after evaluation, so basilar segmentectomy of the left lower lobe was performed using video-assisted thoracoscopic surgery. Grossly, the excised specimen was a gray-red, solitary, well-circumscribed round mass measuring about 3.0 cm in diameter. Microscopically, the tumor was well-defined and composed of cuboidal surface cells and round stromal cells, with mixed multiple growth patterns and consisted of a solid pattern (20% of the total area, **Figure 3A**), a papillary pattern (10% of the total area, **Figure 3B**), a sclerotic pattern (40% of the total area, **Figure 3C**), and a hemangiomatous pattern (30% of the total area, **Figure 3D**). Immunohistochemically, thyroid transcription factor-1(TTF-1), epithelial membrane antigen (EMA), and Vimentin were positive for both cuboidal surface cells and round stromal cells (**Figures 4A-C**). AE1/AE3 was positive in cuboidal surface cells but negative in round stromal cells (**Figure 4D**), while progesterone-receptor (PR) exhibited a negative result in cuboidal surface cells but a positive result in round stromal cells (**Figure 4E**). The Ki-67 index was below 2%, indicating a low proliferation rate (**Figure 4F**). Finally, the pathological and IHC findings of the tumor illustrated the final diagnosis of PSP.

The patient had a total stay length of 12 days. No apparent complications were noted, and the patient recovered well. The patient was followed up for 44 months without recurrence or metastasis until January 2023 (the date of the last follow-up).

## Discussion

PSP was initially referred to as sclerosing hemangioma due to its presumed endothelial and vascular origin. Histologically, PSP is characterized by a distinct pattern of two types of epithelial cells (surface cuboidal cells and stroma round or polygonal cells), forming four architectural patterns: solid, papillary, sclerotic, and angiomatous, which vary in proportions (2, 5, 6). The IHC analysis revealed that TTF-1 and EMA expression in both surface cells and round cells is strongly suggestive of primitive respiratory epithelial derivation (2). Therefore, the World Health Organization (WHO) named it “pulmonary sclerosing pneumocytoma” in 2015 (7). Clinically, PSP is often asymptomatic and is usually detected for unrelated reasons after routine imaging examinations (2). Surgical resection is typically required for an accurate diagnosis and adequate treatment of PSP. However, an accurate radiologic diagnosis is essential, as it determines the surgical approach (8). For patients without clinical symptoms, a follow-up observation strategy is feasible.

In the current case, the old female patient presented solely with a medical history of hypertension and did not exhibit any respiratory symptoms. The hypertension was appropriately managed, and blood pressure levels remained within the normal range. However, the relationship between hypertension and PSP remains unknown. CT is the primary imaging method for diagnosing PSP. Preoperative diagnosis of PSP based on CT findings is often challenging, especially when distinguishing it from other benign tumors, such as pulmonary hamartomas. MRI, as a functional imaging technique, may have the potential to provide additional diagnostic information on PSP. However, the final diagnosis of PSP still relies on the results of pathology and IHC results. The postoperative pathological results showed the tumor contained four characteristic histological patterns. Similar to previous studies (2, 9), the IHC analysis demonstrates positive expression of TTF-1 and EMA markers in surface and round cells, in conjunction with other IHC findings, supporting the final pathological diagnosis of PSP.

Typically, PSP presents as a single, slow-growing, well-circumscribed soft nodule on conventional CT images (4, 10). PSP may exhibit varying degrees of attenuation, corresponding to their histopathological features. It exhibits various enhancement patterns, which may correspond to the varying proportions of its histopathological features and size. While tumors smaller than 3 cm in diameter are likely to exhibit homogeneous enhancement, larger tumors display heterogeneous enhancement (3).

Conventional and advanced MR techniques may provide new insights into the assessment of the various structures of PSP. However, only a limited number of case reports (11–16) have described partial MRI findings of PSP. The relevant literature has been summarized in **Table 1**. On multiple-sequence MRI images, PSP shows heterogeneous SI due to its four distinct internal structures, including solid, papillary, sclerotic, and angiomatous components. In 1995, Han et al. (11) were the first to describe the typical MRI findings of PSP using an MRI scanner. Generally, on T1WI image, the tumor usually revealed as a heterogeneous iso-to-higher SI compared to the thoracic muscle (11, 13, 16). Fujiyoshi et al. (13) speculated that the regions of high SI on the T1WI image corresponded to the solid sclerotic components containing abundant clear cells. On T2WI image, the tumor typically showed markedly high SI with some small low SI foci (12, 13). After intravenous injection of Gadolinium diethylenetriaminepentaacetic acid (Gd-DTPA), the lesion exhibited significant heterogeneous or homogeneous enhancement on fat-suppressed T1WI image (11, 15). High SI areas observed on T2WI image, as well as the noticeable contrast-enhanced areas on post-contrast T1WI, were associated with the presence of an angiomatous component, while slightly elevated intensity areas were thought to indicate the sclerotic component or hemorrhagic components (12, 16). In our case, the tumor presented iso-to-high SI than that of muscle on T1WI and T2WI, which was consistent with previous studies. On fat-suppressed T2-weighted imaging, the corresponding region shows mixed higher SI, highlighting the hemangiomatous region of the tumor. Notably, on the out-phase T1WI image, no local signal reduction was observed (in comparison to the in-phase T1-weighted image), indicating the absence of intratumoral adipose components. Consequently, the diagnosis of pulmonary hamartoma can be excluded. Nakanishi et al. (12) were the first to emphasize the significance of multiphase-sequence enhanced MRI, demonstrating peak enhancement occurring at 2 minutes after gadolinium administration. Kim et al. (16) presented a PSP case demonstrating early enhancement without a distinct peak time, followed by a subsequent plateau pattern. Similarly, the tumor in our case exhibited early and noticeable heterogeneous progressive enhancement without a distinct peak time, followed by a plateau pattern, indicating a benign rather than a malignant lesion. DWI is a noninvasive technique employed for measuring water molecule motion, which can be quantified by using ADC. The ADC values reflect the biological properties of the tissue. Previous studies (17–19) have demonstrated that DWI and ADC measurements can help distinguishing between benign and malignant pulmonary nodules. Mori et al. (14) reported a PSP with a DWI appearance that exhibited a high signal and an ADC value of 1.7×10^-3^mm2/s. This report is the first to present the value of DWI and ADC in diagnosing PSP, but the performance of other MRI sequences was not described. In our case, the tumor showed a high SI on the DWI image, with an ADC value of (2.36 ± 0.22)×10^-3^ mm2/s, indicating a benign tumor. PSP has the potential for growth despite its histopathological benign nature. Kim et al. (16) reported a case of PSP with a wax-and-wane growth pattern. The authors hypothesized that tumor shrinkage and increase in size again were due to intratumoral hemorrhage and resorption. In light of the reports mentioned above, the MRI findings of PSP correspond to its histopathological features, suggesting a benign tumor, but it is not specific.

**Table 1:** Summary of the MRI finding of PSP from the reported cases.

Author(yr)	Gender	Age (years)	Location	Diameter (cm)	Shape	Margin	T1WI	T2WI	Enhancement	Time intensity curve	DWI	ADC (mm^2^/sec)
Han *etal (* 11) (1995)	F	41	in the lateral segment of the right middle lobe.	1.5	round	well-defined	high SI compared with the thoracic muscle	high SI	homogeneous and marked enhancement	NR	NR	NR
Nakanishi *etal (* 12) (1997)	F	63	in the left middle lung field	3	round	well-defined	low SI	slightly high SI	homogeneous enhancement	peak enhancement was seen 2 minutes after the administration of Gd-DTPA	NR	NR
Fujiyoshi *etal (* 13) (1998)	M	30	in the left lower lobe of lung	NR	oval	well-defined	heterogeneous SI including higher and lower SI than that of muscle	heterogeneous high and higher SI centrally with peripheral areas of lower SI than that of adipose	heterogeneous enhancement; the central portion of the mass showed intense enhancement	NR	NR	NR
Fujiyoshi *etal (* 13) (1998)	F	72	in the right lower lobe of lung	NR	round	well-defined	high SI nearly equal to that of adipose	higher SI than that of adipose tissue with some low SI foci	enhanced totally	NR	NR	NR
Mori *etal (* 13) (2010)	F	33	in the hilum of the upper lobe of left lung	1.9	round	well-defined	NR	NR	NR	NR	high SI	1.7 × 10^-3^
Kuroda *etal (* 15) (2012)	F	61	in the hilum of the upper lobe of left lung	7.0	round	well-defined	NR	NR	heterogeneous enhancement	NR	NR	NR
Kim *etal (* 16) (2015)	F	47	in the upper lobe of left lung	3.1	round	well-defined	iso- to higher SI than that of muscle	heterogeneously high SI on fat-saturated T2WI image	homogeneous enhancement	early enhancement without peak point and subsequent plateau pattern	NR	NR

M, male; F, female; NR, not report; yr, year; SI, signal intensity.

Various similar benign or malignant pulmonary tumors should be considered in the differential diagnosis of PSP. The most important differential diagnosis for PSP is pulmonary hamartoma, which is the most common benign pulmonary tumor. However, some pulmonary hamartomas do not exhibit the characteristic popcorn-like calcification on CT scans or visible fat during gross examination. These types of hamartomas also present with contrast enhancement. Chemical shift MRI is a valuable tool for identifying intracellular lipids, making it helpful in detecting fatty components within pulmonary hamartomas (20, 21). On the out-phase T1WI image, a localized signal reduction can be observed within the mass compared to the in-phase T1WI image. After a contrast-enhanced scan, hamartoma usually has a higher prevalence of network enhancement (22). Pulmonary tuberculosis granuloma is another common benign lesion in the lungs. In contrast to PSP, pulmonary tuberculosis granuloma typically exhibits lower enhancement levels, often with rim enhancement (22). On the other hand, PSP usually displays noticeable enhancement throughout the entire tumor (13). Pulmonary malignant tumors, on the other hand, are characterized by prominent lobulation with spiculated margins (23), a significant decrease in ADC values (24), and marked heterogeneous enhancement. These characteristics help differentiate them from PSP.

## Conclusion

In summary, the MRI findings of PSP in our case were consistent with those described in the existing literature. More importantly, we presented a more comprehensive MRI findings of PSP in multiple sequences, such as in-phase and out-phase T1WI, T2WI, fat-suppressed T2WI, DCE, DWI, and ADC. These findings will be valuable for diagnosing PSP and provide new diagnostic clues for differentiation. However, it is important to note that a broad consensus on MRI findings for PSP has not yet been reached. Therefore, conducting studies with a larger number of cases is our future research direction to improve diagnostic accuracy.

## Data availability statement

The original contributions presented in the study are included in the article/supplementary material. Further inquiries can be directed to the corresponding author.

## Ethics statement

The studies involving humans were approved by Ethical Review Board of the Fourth Hospital of Hebei Medical University. The studies were conducted in accordance with the local legislation and institutional requirements. The patients/participants provided their written informed consent to participate in this study. Written informed consent was obtained from the individual(s) for the publication of any potentially identifiable images or data included in this article.

## Author contributions

YL, XG, NZ, and YW wrote the manuscript. HD undertook the pathological analysis of the specimens. HF, QW and GS undertook the radiological diagnosis. MW provided the clinical and surgical data of the patient. All authors contributed to the article and approved the submitted version.
